# From Piezoelectric Nanogenerator to Non-Invasive Medical Sensor: A Review

**DOI:** 10.3390/bios13010113

**Published:** 2023-01-09

**Authors:** Qiliang Zhu, Tong Wu, Ning Wang

**Affiliations:** 1 Center for Green Innovation, School of Mathematics and Physics, University of Science and Technology Beijing, Beijing 100083, China; 2 National Institute of Metrology, Beijing 100029, China

**Keywords:** piezoelectric nanogenerator, non-invasive sensor, medical application, self-powered sensing system

## Abstract

Piezoelectric nanogenerators (PENGs) not only are able to harvest mechanical energy from the ambient environment or body and convert mechanical signals into electricity but can also inform us about pathophysiological changes and communicate this information using electrical signals, thus acting as medical sensors to provide personalized medical solutions to patients. In this review, we aim to present the latest advances in PENG-based non-invasive sensors for clinical diagnosis and medical treatment. While we begin with the basic principles of PENGs and their applications in energy harvesting, this review focuses on the medical sensing applications of PENGs, including detection mechanisms, material selection, and adaptive design, which are oriented toward disease diagnosis. Considering the non-invasive in vitro application scenario, discussions about the individualized designs that are intended to balance a high performance, durability, comfortability, and skin-friendliness are mainly divided into two types: mechanical sensors and biosensors, according to the key role of piezoelectric effects in disease diagnosis. The shortcomings, challenges, and possible corresponding solutions of PENG-based medical sensing devices are also highlighted, promoting the development of robust, reliable, scalable, and cost-effective medical systems that are helpful for the public.

## 1. Introduction

In recent years, we have increasingly sought new technologies to monitor and improve our health [1,2,3] and physiological performance [4,5]. As a result, wearable sensors for measuring human physiologic parameters in a continuous [6,7], non-intrusive [8,9], and real-time manner [10,11] are undergoing quick progress. By integrating with chemical sensing technology, wearable sensors are gaining momentum, accelerating their expansion as replacements for the expensive analytical instruments in the health care industry due to their low cost, simplicity, and portability [12,13,14,15]. 

In comparison with conventional medical sensors, wearable biosensors are expected to be able to measure analytes from eccrine sweat instead of biomarkers in vivo to assess the health condition and body vitality; thus, they are deemed as the next step in effective detection [16,17,18]. With the help of information technology, such as Bluetooth connectivity, these wearable techniques even have the potential to simultaneously complete tasks such as data collection, physiological parameter tracking, and online analysis and provide real-time health monitoring solutions to us, dependent on whether they can meet the requirements for accuracy [19,20,21,22]. Consequently, the patients are free of risk of infection and can receive care in vitro, even when multiple readings are required at regular intervals [23,24,25]. However, for mobile clinical testing, the current power sources, such as electrochemical batteries, usually face challenges related to their volumetric energy density capacity, which is key to meeting the requirements for sustainable operation [26] and realize the full power of wearable electronics [27]. Currently, renewable energy harvesting technologies such as piezoelectric nanogenerators (PENG) [28], triboelectric nanogenerators (TENG) [29,30], pyroelectric nanogenerators (PyNG)/thermoelectric generator (TEG) [31], and solar cells [32] are attracting great attention due to their potential to complement electrochemical power sources.

PyNG-/TEG-based sensors are more sensitive to temperature, but they can only collect thermal energy generated by the temperature difference between humans and the environment through the Seebeck effect, thus providing power to medical sensing devices, and solar cells can only convert energy from sunlight or indoor light into electrical energy by the photovoltaic effect. PENG and TENG have a relatively wide range of energy sources, ranging from basic daily movements to physiological activities [33,34]. In addition, PENG can operate without a contact separation process of the two materials, and the piezoelectric material possesses a dielectric constant, low dielectric loss, and high electromechanical coupling coefficient [35]. These advantages render PENG a suitable candidate for medical sensor power supplies. However, in operation, PENGs typically experience different mechanical and electrical loading conditions that can deteriorate their electromechanical properties, rendering them unsuitable for specific applications [36]. Microstructural design, such as the tuning of domain configurations, can improve the mechanical and piezoelectric properties of piezoelectric materials [37].

PENG was initially invented as an energy-harvesting device with a stable output, high interference immunity, high reliability, and low leakage current loss [38,39,40]. To date, extensive research has been carried out in various aspects, such as mechanism exploration [41], structure design [42], performance optimization [43], material selection [44,45,46], and their various applications in health monitoring devices [47,48]. Various piezoelectric materials, such as ZnO and PZT, have been extensively studied according to their electromechanical coupling characteristics and their microstructural features, which paved the way for the biosensing application of PENG in the biomedical field. Furthermore, abundant medical information about a patient’s health status can be received through the wearing or implantation of self-powered sensors [49]. Meanwhile, by harvesting energies from various biomechanical actions, including breathing, movement, the heartbeat, pulse, blood flow, and vascular pressure [50,51,52,53], the generated piezoelectric output also provides copious chemical or biological information, which can be used for the development of PENG-based medical sensor systems [54,55,56].

**Figure 1:**
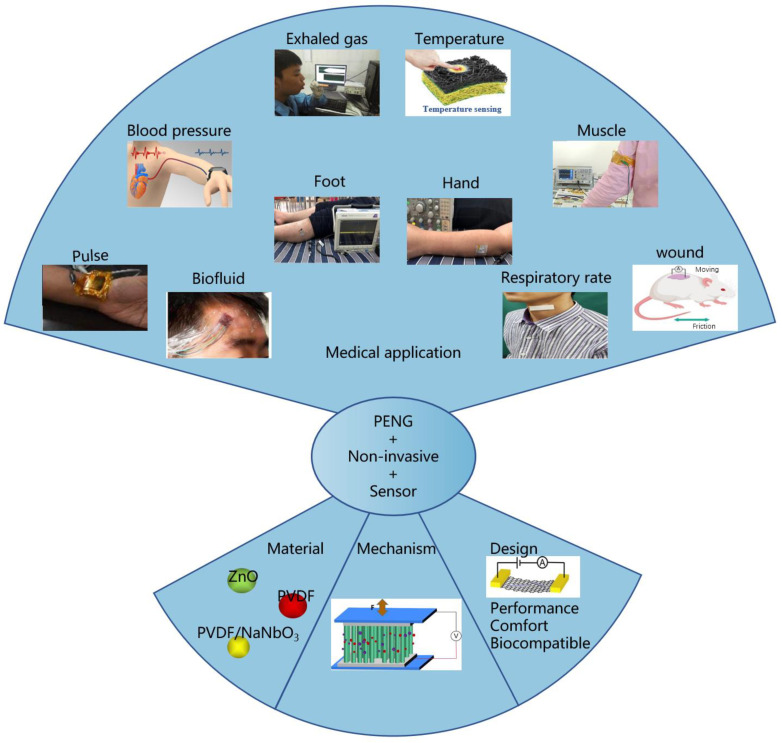
Recent progress in PENG-based non-invasive medical sensors, *F* is force, *A* is current meter, and *V* is voltmeter.

Herein, the recent advancements in PENG-based medical sensors are reviewed and established. As shown in Figure 1, PENG-based medical sensor systems can be classified as either self-powered non-invasive sensors or self-powered non-invasive biosensors on the basis of the role of the sensor function. Accordingly, the design criteria, material selection, and fundamentals of PENG-based chemical sensors are discussed in terms of their high performance, high sensitivity, reliability, biocompatibility, miniaturization, and multifunctionality. For the sake of simplicity, PENG-based medical sensors are categorized into two main groups according to their daily applications: mechanical sensors and biosensors. The former are placed in more active and powerful positions, thus possessing a higher output performance, while the latter are in direct contact with biomarkers and possess a higher sensing performance. Finally, the challenges and prospects for the advancement of PENG-based medical sensors are briefly discussed. We hope that this review will contribute to the design and operation of efficient and sustainable PENG-based medical sensor systems.

## 2. Basic Principle of PENG

### 2.1. Piezoelectric Effect

According to previous studies, the pressure and generated charge have a strong linear relationship with the piezoelectric effect. The positive and negative charge centers are relatively displaced when the piezoelectric material is distorted by an external force. The differential in the displacement of charge centers creates polarization inside the material if the piezoelectric material is electrically neutral overall. Meanwhile, charges of opposite polarity manifest themselves on the piezoelectric material’s surface at both ends. It reverts to its uncharged form when the external force is no longer present. This is the benefit of piezoelectricity. The transfer of positive and negative charges and the deformation of the piezoelectric material, however, occur when the piezoelectric material is exposed to an external electric field. The dielectric no longer deforms when the electric field is removed. This phenomenon, i.e., the inverse piezoelectric effect, is also named the electrostriction phenomenon [57,58]. 

There are four main types of materials with piezoelectric effects: piezoelectric single crystals (ZnO, CdS), piezoelectric polycrystals (BaTiO_3_, PZT), piezoelectric polymers, and piezoelectric composites [59].Taking ZnO as an example, PENGs fabricated from n-type conductive ZnO nanowires and Au can form Schottky barriers. The Schottky barrier is a rectification contact that only allows electrons to move from ZnO to Au. The internal and exterior free carriers somewhat balance out the polarized charge that forms at the tips of the stressed ZnO wire. In a steady state, the two contact points still have a piezoelectric charge. The positive charge’s electrostatic field causes the Schottky barrier’s height to decrease, whereas the negative charge causes it to increase. They create an asymmetric barrier that renders it easier for the electrons to move [60]. In addition, the theory suggests that the strain is related to the change in the Schottky barrier height, which is proportional to the piezoelectric charge density. Since the current is proportional to the Schottky barrier height, the strain is proportional to the current flow [61]. Moreover, the piezoelectric effect is not a fleeting phenomenon. The piezoelectric effect persists as long as the strain is constant because of the residual piezoelectric charge at the interface [62]. Environments such as atmosphere and humidity can affect the Schottky barrier, rendering it difficult to achieve a stable, repeatable, and uniform Schottky contact under various circumstances. Therefore, creating repeatable and stable Schottky connections at the metal–ZnO interface is the first challenge to overcome in the design of PENGs. 

### 2.2. Working Principle

**Figure 2:**
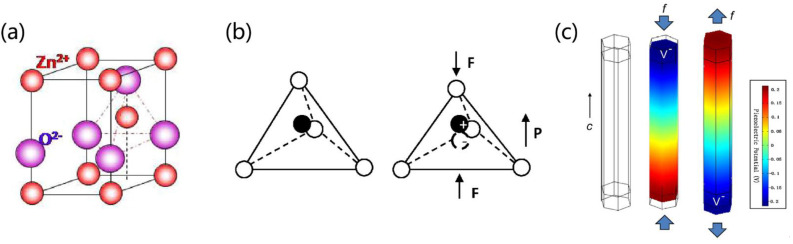
Principle and charge output process of the piezoelectric nanogenerator. (**a**) Model of ZnO’s atomic structure. (**b**) The material’s piezoelectric characteristics and the various piezoelectric potentials under compression and tension, *F* is the force and *P* is the piezoelectric potential (piezopotential). Reprinted with permission from [64]. Copyright 2010, Elsevier. (**c**) Diagram of the piezoelectric potential distribution of ZnO, *c* is the c-axis, which is the growth direction of ZnO nanowires, *f* is the force, and *V* is the piezoelectric potential. Reprinted with permission from [65]. Copyright 2009, AIP Publishing.

A PENG is a device that uses piezoelectric material as an electrode to generate an electrical potential difference or an external circuit design in order to accumulate and release an electrical charge [63]. The basic principle of electricity generation from piezoelectric material is the breaking of the centrosymmetry of the crystal structure under the action of an external force, resulting in the formation of a piezoelectric potential. For example, taking a common zinc oxide (ZnO) crystal as an example, as shown in Figure 2a, the tetrahedral coordination of Zn^2+^ and O^2−^ accumulates along the c-axis. In the initial state, the centers of the Zn^2+^ cation and the O^2−^ anion overlap. Once a strain is applied along the c-axis, the centers of the Zn^2+^ cation and O^2−^ anion are misaligned, inducing an electric dipole that leads to a piezoelectric potential in ZnO, which drives the flow of free electrons in the external circuit (Figure 2b) [64]. When strain is applied to ZnO along the c-axis, its two sides can generate positive and negative piezoelectric potentials, respectively (Figure 2c) [65]. By periodically exerting dynamic external forces, the piezoelectric potential can be changed sequentially, which helps to replace the continuous flow of pulsed current through the external circuit.

## 3. Recent Progress in PENG-Based Non-Invasive Medical Sensors

In recent years, the advancement in the creation of stable, reliable, stretchable, flexible, and high-performance piezoelectric materials has provided medical sensors with extensive opportunities. PENG-based sensors can convert biomechanical energy from body motions such as respiration, blood circulation, gastrointestinal motility, and movement into electrical output, which, in turn, can be used to monitor physiological health conditions [66,67,68]. As a result, PENG-based sensors can work as a power source and active sensor simultaneously, meeting the increasing need for autonomous and sustainable medical devices. This section details the basic principles, material selection, and design strategies of, as well as the recent advances in, PENG-based non-invasive sensors.

### 3.1. Sensing Mechanism

**Figure 3:**
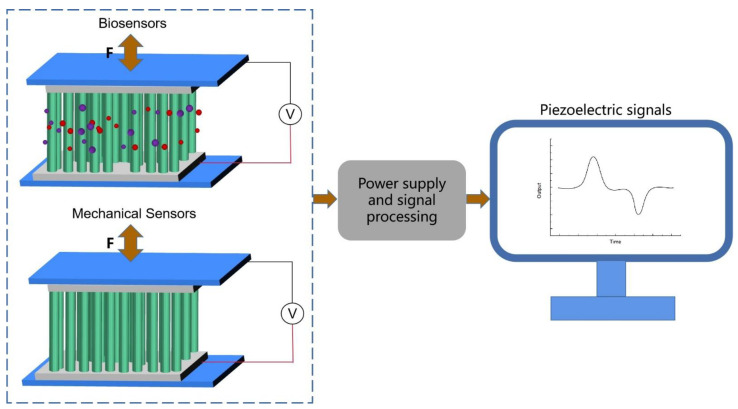
Sensing mechanism of PENG-based sensors, *V* is the voltmeter, *F* is the force.

The distinctive properties of piezoelectric materials have driven their rapid development in many fields, among which sensing technology based on the piezoelectric effect is one of the most fruitful areas. At the root, the piezoelectric effect is due to the mechanical stress in the lattice of a piezoelectric material that changes the distance between the center of the positive and negative charges, thus changing the original dipole moment and resulting in a polarized charge on the surface of the piezoelectric material. For PENGs, there are two main sensing mechanisms, as shown in Figure 3.

One type of sensing is mechanical, monitoring motions, the respiratory rate, and pulse rate, to name a few examples. After the device is attached to a person, the PENG is pulled and bent when subjected to mechanical forces, and its upper surface experiences tensile stress. Meanwhile, the lower surface of the PENG experiences compressive stress, which deforms the piezoelectric layer, thereby generating a polarized charge. The resulting electric field attracts or repels electrons in the electrodes, and an electrical signal, the response of the mechanical force to the PENG, is generated when an external circuit is connected in order to allow the generated electrons to move from the electrode surface to the bottom electrode. Due to the flexibility of the PENG, it will return to its initial state once the external force is removed. Once the stress state is reversed, the electrons gathered at the bottom electrode will return to the top electrode, generating an opposite electrical pulse. Thus, the compression and release of the PENG can generate cyclic alternating piezoelectric signals during human movement, respiration, and in the pulse. In addition, the layered baklava structure is sensitive to stress changes. This sensor does not involve biochemical reactions and relies on the physical properties of the material itself. Another type is biosensing, monitoring aspects such as body fluids and gas molecules. Owing to the coupling of the piezoelectric effect and molecular sensitivity properties, the piezoelectric output of the PENG can served as both a power source and a sensing signal. The surface of the piezoelectric material has a high density of point defects, such as oxygen vacancies, which provide adsorption sites for the target molecules and form an adsorption layer [69]. When subjected to an external force, the material surface can generate a high local charge density and a strong electrostatic field, which dissociates the adsorbed molecules into ions. The ion, in turn, releases a proton to the neighboring molecule, thus resulting in the charge transfer generated by the Grotthuss chain reaction [70]. Overall, when the PENG is compressed and deformed, both the ions in the adsorbed layer and the electrons inside the piezoelectric material can move in a directional manner, shielding the polarized charge on the surface of the piezoelectric material and thus reducing the piezoelectric output of the device [71,72,73].

The electromechanical coupling behavior of piezoelectric materials can be described by the following equation:

(1) $$T_{ij}=c_{ijkl}^{E}S_{kl}-e_{kij}E_{k}$$

(2) $$D_{i}=e_{ikl}S_{kl}+k_{ij}^{s}E_{k}$$

where *S*, *T*, *D*, and *E* are the mechanical strain, mechanical stress, potential shift, and electric field, respectively. $e_{kji}$, $k_{ij}^{S}$, and $c_{ijkl}^{E}$ are the piezoelectric constant, dielectric constant, and elastic constant, respectively. The superscript “*E*” indicates a constant electric field, and “*S*” indicates a constant strain [74]. Equation (1) describes the inverse piezoelectric effect, i.e., the strain or displacement that occurs when an electric field is applied to a piezoelectric material. Equation (2) describes the direct piezoelectric effect, where an electrical charge is generated when an external force is applied to the material. This effect is used for sensing and energy harvesting. Equations (1) and (2) can be represented explicitly in matrix form:

(3) $$\left[\begin{array}{c} \sigma_{11}\\ \sigma_{22}\\ \sigma_{33}\\ \sigma_{23}\\ \sigma_{31}\\ \sigma_{12} \end{array}\right]=\left[\begin{array}{cccccc} c_{11} & c_{12} & c_{13} & 0 & 0 & 0\\ c_{12} & c_{22} & c_{13} & 0 & 0 & 0\\ c_{13} & c_{13} & c_{33} & 0 & 0 & 0\\ 0 & 0 & 0 & c_{44} & 0 & 0\\ 0 & 0 & 0 & 0 & c_{55} & 0\\ 0 & 0 & 0 & 0 & 0 & c_{66} \end{array}\right]\left[\begin{array}{c} \varepsilon_{11}\\ \varepsilon_{22}\\ \varepsilon_{33}\\ 2\varepsilon_{23}\\ 2\varepsilon_{31}\\ 2\varepsilon_{12} \end{array}\right]-\left[\begin{array}{ccc} 0 & 0 & e_{31}\\ 0 & 0 & e_{32}\\ 0 & 0 & e_{33}\\ 0 & e_{24} & 0\\ e_{15} & 0 & 0\\ 0 & 0 & 0 \end{array}\right]\left[\begin{array}{c} E_{1}\\ E_{2}\\ E_{3} \end{array}\right]$$

(4) $$\left[\begin{array}{c} D_{1}\\ D_{2}\\ D_{3} \end{array}\right]=\left[\begin{array}{cccccc} 0 & 0 & 0 & 0 & e_{15} & 0\\ 0 & 0 & 0 & e_{24} & 0 & 0\\ e_{31} & e_{32} & e_{33} & 0 & 0 & 0 \end{array}\right]\left[\begin{array}{c} \varepsilon_{11}\\ \varepsilon_{22}\\ \varepsilon_{33}\\ 2\varepsilon_{23}\\ 2\varepsilon_{31}\\ 2\varepsilon_{12} \end{array}\right]-\left[\begin{array}{ccc} k_{11} & 0 & 0\\ 0 & k_{22} & 0\\ 0 & 0 & k_{33} \end{array}\right]\left[\begin{array}{c} E_{1}\\ E_{2}\\ E_{3} \end{array}\right]$$

where *σ* denotes mechanical stress, *ε* is the strain, and *k* denotes the dielectric constant under constant strain. Since the elastic, piezoelectric, and dielectric constants depend on other constants, many piezoelectric materials can be understood as transversely isotropic.

### 3.2. Material Selection of PENGs for Energy Harvesting and Sensing

The applications of PENGs for sensing and energy harvesting have expanded rapidly over the last few decades. Most of the reported works are based on inorganic piezoelectric materials, such as zinc oxide, or other materials, such as polymers, with high voltage electrical coefficients, and composites are also widely used. This section provides an overview of the selection of piezoelectric materials and the application of piezoelectric thin films to achieve a cycle of piezoelectric materials at different length scales, ranging from bulk to nano.

#### 3.2.1. Inorganic Piezoelectric Materials

The most widely used inorganic piezoelectric materials include ZnO, PZT, BaTiO_3_, MoS_2_, and BiFeO_3_. Zinc oxide is an excellent piezoelectric material and is commonly used to fabricate sensor devices and transistors. The flexibility, biosafety, and piezoelectric effect render ZnO a promising candidate for non-invasive sensors [75,76]. 

**Figure 4:**
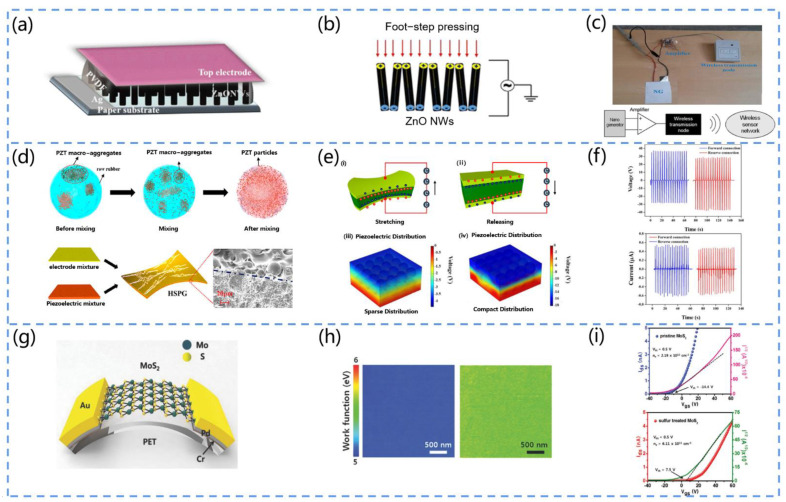
(**a**) Structure diagram of the ZnO-based PENG. (**b**) Graph of the voltage profile generated by the PENG under foot pressure. (**c**) The complete operation schematic of the device. Reprinted with permission from [77]. Copyright 2016, SpringerOpen. (**d**) Schematic diagram of the fabrication process and structure of the PZT-based piezoelectric nanogenerator (HSPG). (**e**) Schematic diagram of the operation of the HSPG (i,ii) and the simulation of the piezoelectric potential distribution when the PZT is sparsely or tightly distributed throughout the substrate (iii,iv). (**f**) Plots of the output of the forward- and reverse-connected HSPG. Reprinted with permission from [78]. Copyright 2019, ACS Publications. (**g**) Schematic diagram of the PENG structure based on MoS_2_ nanosheets. (**h**) Comparison of the KPFM images of MoS_2_ nanosheets and S-treated MoS_2_ nanosheets. (**i**) *I_ds_-V_gs_* plots of MoS_2_ nanosheets and S-treated MoS_2_. The voltage is shifted and the carrier density is significantly reduced. Reprinted with permission from [80]. Copyright 2018, Wiley Online Library.

For example, Nour et al. proposed a piezoelectric nanogenerator (PENG) based on zinc oxide (ZnO) nanowires (Figure 4a) [77]. They prepared the PENG by hydrothermally growing ZnO nanowires on a paper substrate. The device can harvest energy from low-frequency motion (footsteps) for self-powered pressure sensors (Figure 4b,c).

Owing to its low cost, high piezoelectric performance, and stable thermal properties, PZT is widely applied. Niu et al. developed a high-performance PZT-based stretchable piezoelectric nanogenerator (HSPG) (Figure 4d) [78]. They adopted a mixing technique instead of the conventional stirring technique to blend the PZT into silicone rubber, which greatly improved the uniformity of the PZT distribution in the matrix (Figure 4e). The tensile rate was increased to 30%, and the output performance was significantly improved (Figure 4f). The device can be conformally attached to the body to obtain kinetic energy by deformation.

As lead is toxic and can pollute the environment and harm health, some lead-free piezoelectric materials have been developed. For example, Baek et al. designed a lead-free PENG based on BaTiO_3_ nanowire arrays (BaTiO NWs) [79]. They adopted a two-step hydrothermal method to grow BaTiO NWs on a Ti substrate. To measure the piezoelectric energy of the individual BaTiO_3_ single grains, they transferred the NWs to a flexible substrate and connected the NWs to a Au electrode pad. When the device was pressurized with a finger, the output voltage and current values were 90 V and 1.2 µA, respectively.

The MoS_2_ with semiconducting and piezoelectric properties may also serve as a candidate material for piezoelectric devices. Han et al. developed a MoS_2_-based PENG (Figure 4g) [80]. They fabricated S-treated monolayer MoS_2_ nanosheets on a sapphire substrate by the CVD method and then transferred them to PET. They compared the performance of the PENG before and after the S treatment, and the results showed that the piezoelectric output of the s-treated MoS_2_-based PENG was significantly increased. This can be attributed to the fact that the S treatment process effectively passivates the S vacancies, reduces the free carriers, and prevents the shielding effect (Figure 4h,i). Therefore, the piezoelectric output of the S-treated MoS_2_-based PENG was significantly increased compared to the original MoS_2_.

BFO possesses excellent physical properties but is one of the least studied lead-free chalcogenide materials used for piezoelectric applications. Sankar et al. fabricated a BiFeO_3_ (BFO) by the sol–gel method and obtained spherical BFO nanoparticles after annealing at 100–500 °C [81]. Then, they prepared a sandwich structure piezoelectric device (graphene/BiFeO_3_-PDMS/graphene). The output voltage of the device could reach 0.4 V when finger pressure was exerted.

Due to the huge market demand for piezoelectric products, piezoelectric materials and devices are advancing rapidly and exhibiting a higher biocompatibility, piezoelectric properties, and transconductance efficiency compared to conventional piezoelectric materials, such as PZT and BT. In addition, piezoelectric metamaterials are a strategy that can be used to achieve high-performance, lead-free piezoelectricity. Ceramic sheets can be transformed into piezoelectric metamaterials by chemical inhomogeneities, which may be caused by the formation and diffusion of oxygen vacancies during the reduction process. Furthermore, the novel stretchable structures of inorganic piezoelectric films have been explored in order to render them stretchable, with wavy and flexural geometries being examples.

#### 3.2.2. Piezoelectric Polymers

Piezoelectric polymers are carbon-based materials. Owing to their specific molecular structure and orientation, they can exhibit piezoelectricity. They are softer than ceramic materials and possess the advantages of simplicity, flexibility, and a low density for energy harvesting. Polymers with a semi-crystalline structure can exhibit piezoelectricity due to the microscopic crystals distributed in the amorphous body, including PVDF, P(VDF-TrFE), cellulose, PLA, polyamides, paraxylene, etc. [82,83]. Piezoelectricity can also be observed in amorphous or non-crystalline polymers, resulting from the presence of dipoles in their molecular structure, which form at temperatures above the glass transition temperature (Tg) of the polymer. Such polymers include nylon, polyimide, polyurethane, polyurea, etc. [84].

**Figure 5:**
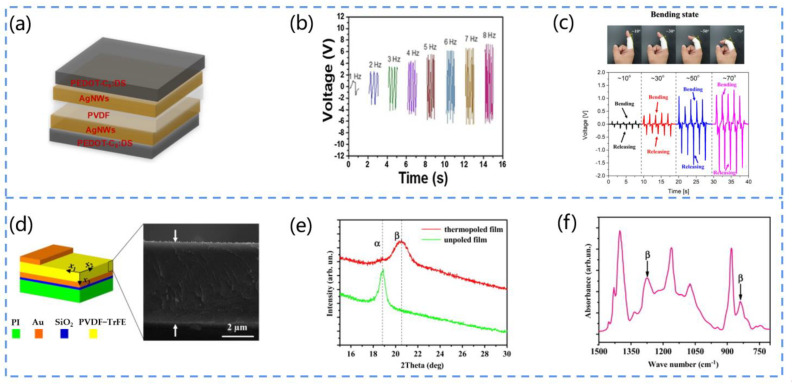
(**a**) Schematic diagram of the structure of a PVDF-based PENG. (**b**) Voltage output of the PENG at 1~8 Hz frequency. (**c**) Performance of the PENG under the movement of the tester’s index finger. Reprinted with permission from [86]. Copyright 2019, Elsevier. (**d**) Schematic diagram of the structure of a PVDF-TrFE-based PENG, the arrows correspond to 6.5μm thick PVDF-TrFE film. (**e**) The X-ray diffraction pattern of the PVDF-TrFE film, α corresponds to the α phase in the unpolished film and β corresponds to the β phase in the thermopolymer film. (**f**) FTIR spectra of PVDF-TrFE films, the arrows correspond to the infrared characteristic peaks of the β-phase of the film. Reprinted with permission from [87]. Copyright 2014, Elsevier.

PVDF and P(VDF-TrFE) are the most commonly used piezoelectric polymers [85]. Owing to their piezoelectricity, flexibility, and biocompatibility, they have great potential for medical sensing. There are α, β, γ, and & phases in PVDF, in which the β phase is closely related to the piezoelectricity. Moreover, the α phase can transform into the β phase by stretching. For example, Khadtare et al. fabricated a flexible piezoelectric nanogenerator for the real-time monitoring of muscle activity using PVDF film as the active layer (Figure 5a) [86]. The device had an excellent flexibility, stability, and transparency. The output voltage and current could reach 7.02 V and 1.11 μA, respectively, during the stretch–release process at 8 Hz (Figure 5b). In addition, the device could be adhered to the finger in order to monitor human muscle movement through the piezoelectric response obtained from finger flexion and release (Figure 5c).

Pi et al. developed a flexible piezoelectric nanogenerator (PENG) using polyvinylidene fluoride-trifluoroethylene (PVDF-TrFE) film as a functional layer (Figure 5d) [87]. They prepared the PVDF-TrFE film by the spin-coating method and used it as the active layer of the PENG. Furthermore, they polarized the PENG in the vertical surface direction at 100 °C to induce the electric dipole moment in order to align it with the electric field direction. The results indicated by the XRD and IR spectra showed a high content of β phase in the thermopolar copolymer film, and the enhancement of the piezoelectric output performance originated from this (Figure 5e,f).

#### 3.2.3. Piezoelectric Polymer Nanocomposites

By adding fillers with high voltage electrical properties to polymers, it is possible to obtain piezoelectric polymer composites with their advantages, which are the high voltage electrical constants of the fillers and flexible properties of the polymers.

**Figure 6:**
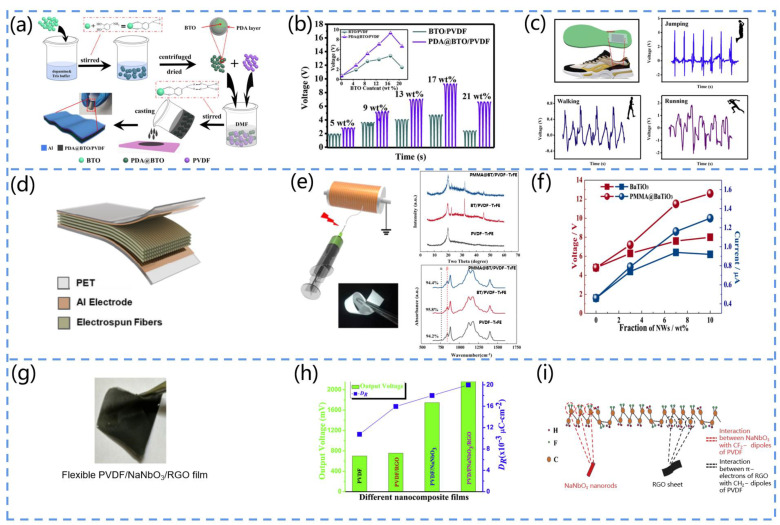
(**a**) Illustration of the preparation process of the PDA@BTO/PVDF composite membrane. (**b**) Output profiles of the BTO/PVDF and PVDF/PDA@BTO-based sensors when the BTO content is varied. (**c**) Voltage response to running, walking, and jumping upon embedding in the insole. Reprinted with permission from [88]. Copyright 2020, Elsevier. (**d**) Schematic diagram of the structure of PENG. (**e**) The process of electrostatic spinning to fabricate composite fibers and their XRD spectra and FT-IR spectra, the α corresponds to the nonpolar α-phase of PVDF-TrFE and the β corresponds to the electroactive β-phase of PVDF-TrFE. (**f**) Output voltages and currents of PENGs with different NWs components. Reprinted with permission from [89]. Copyright 2021, Elsevier. (**g**) Diagram of the structure of the PVDF/NaNbO_3_/RGO composite membrane. (**h**) Output voltage and residual polarization of the PVDF/NaNbO_3_/RGO composite membrane. (**i**) Mechanistic diagram of the interaction of NaNbO_3_ and RGO with PVDF (H, F, and C correspond to the hydrogen, fluorine, and carbon elements, respectively). Reprinted with permission from [90]. Copyright 2017, Elsevier.

Yang et al. fabricated a piezoelectric pressure sensor based on PDA@BTO/PVDF film [88]. They first modified barium titanate (BTO) with polydopamine (PDA), homogeneously mixed it with polyvinylidene fluoride (PVDF), and, finally, prepared the piezoelectric sensor by the surface solution casting method (Figure 6a). The voltage output was significantly enhanced by 13.3 times in comparison to the original PVDF, exhibiting a promising power supply capability (Figure 6b). Furthermore, the signals of joint flexion and human motion could be monitored, and this study showed that the composite film has great potential for application in human wearable devices (Figure 6c).

Shi et al. developed a flexible BaTiO_3_/PVDF-TrFE-based PENG (Figure 6d) [89]. They fabricated PMMA@BaTiO_3_ NWs by the atom transfer radical polymerization (ATRP) method and used them as the piezoelectric reinforced phase to prepare PVDF-TrFE nanocomposites by the electrostatic spinning method (Figure 6e). The compatibility of PVDF-TrFE with the PMMA material greatly improved the dispersion of the BaTiO_3_ nanowires and stress transfer at the interface, thus significantly improving the output performance of the PENG (Figure 6f).

Singh et al. fabricated a PVDF/NaNbO_3_/RGO flexible composite film and investigated its piezoelectric response (Figure 6g) [90]. The results indicated that it had the same degree of β phase as the bare PVDF, but the nanogenerator based on the composite film had a higher voltage signal (output voltage of 2.16 V and current of 0.383 a) (Figure 6h). This result originated from the presence of NaNbO_3_ and RGO, which enabled the easier alignment of the dipoles of the PVDF (Figure 6i). Moreover, the piezoelectric properties of NaNbO_3_ itself were also responsible.

### 3.3. Design Criteria of PENG-Based Non-invasive Sensors

#### 3.3.1. Performance Improvement Strategy

The goal of improving the performance of PENG-based sensors is mainly to improve the flexibility, sensitivity, and piezoelectricity of the device. The commonly employed methods include the use of materials with high voltage coefficients, the development of composite thin film materials, changing the micromorphology of the material, the addition of dopants, and the selection of a suitable substrate. Materials with high voltage electrical coefficients and composite thin film materials have been mentioned previously, and the remaining methods are comprehensively analyzed in this section to provide a guide for the development of high-performance PENG-based non-invasive sensing devices.

##### Micromorphology

By controlling the process conditions, different micromorphological materials such as nanowires, nanoribbons, nanorods, nanotubes, etc., can be prepared. Different micromorphologies of the same piezoelectric material have great impacts on the performance of piezoelectric sensors [91].

**Figure 7:**
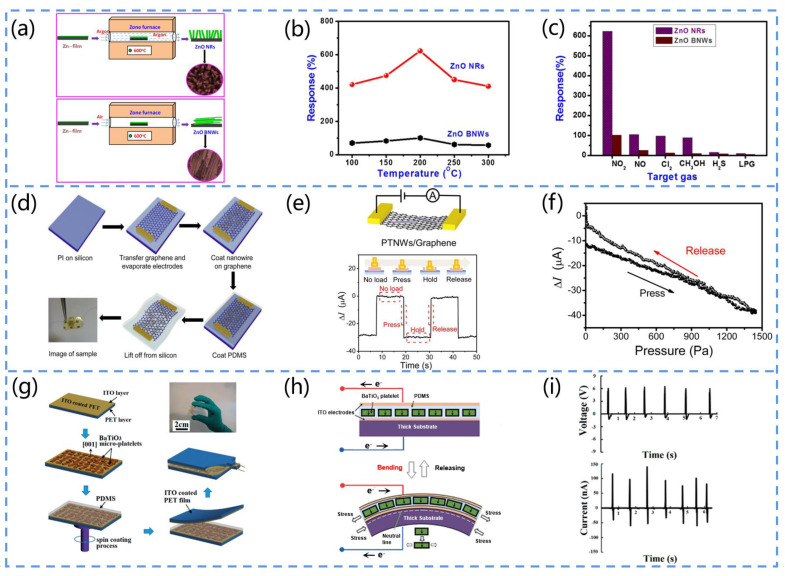
(**a**) Fabrication process of ZnO NR and ZnO BNW. (**b**) Responses of ZnO sensors at different temperatures. (**c**) Selectivity of ZnO sensors for NO_2_ gas. Reprinted with permission from [92]. Copyright 2017, Elsevier. (**d**) Manufacturing process of PTNW/graphene-based pressure sensors. (**e**) Pressure responses of PTNWs/graphene under pulsed pressure (*A* is the current meter). (**f**) Variation in the current of PTNW/graphene-based pressure sensors during pressure release. Reprinted with permission from [95]. Copyright 2017, ACS Publications. (**g**) Process diagram of the BaTiO_3_-based PENG fabricated using PET substrate. (**h**) The subplots are the cross-sectional views of the PENG when operating in bending and release modes, respectively. (**i**) The subplots present the output voltage and current of the PENG when the finger is bent, respectively. Reprinted with permission from [97]. Copyright 2015, the Royal Society of Chemistry.

Navale et al. prepared graded ZnO nanowires (BNWs) and nanorods (NRs) on glass substrates by the surface thermal evaporation method (Figure 7a) [92]. The prepared ZnO NRs and BNWs were used as sensing materials for the detection of toxic nitrogen dioxide (NO_2_). The ZnO sensors had a high response to NO_2_, with a maximum response of 622 and 101% and fast response and recovery time values at 200 °C (Figure 7b,c). Moreover, the ZnO sensor responded to very low exposure to NO_2_ gas (1 ppm). The excellent stability and reproducibility of the response enables ZnO with nanostructures to be widely applied in the development of wearable sensors.

Koka et al. synthesized a BaTiO_3_ nanowire array that can be applied for vibration sensing and vibration energy harvesting [93]. They adopted a two-step hydrothermal reaction method to control the growth of titanate NW arrays and then convert the titanate NW arrays into BaTiO_3_ NW arrays as precursors, while maintaining the nanowire shape. The arrays possessed a tetragonal phase, thus exhibiting ferroelectric and piezoelectric properties. At a lower resonant frequency (170 Hz), the BaTiO_3_ NWs exhibited a superior output voltage response of up to 345 mV when excited by sine-wave-based acceleration.

##### Dopants

To improve the properties of sensing devices, various dopants have been developed, such as metallic elements, graphene, etc. Their addition to piezoelectric materials can significantly increase the piezoelectric coefficient (d33) [94].

Chen and his team designed a graphene-based pressure sensor (Figure 7d) [95]. The device exhibited a sensing behavior for static signal measurements that originated from the piezoelectric-potential-induced transient electron flow (Figure 7e). Furthermore, owing to the high carrier mobility of the graphene, the device possessed a high sensing performance (sensitivity of up to 9.4 × 10^−3^ kPa^−1^, response time of 5–7 ms) and excellent flexibility, making it suitable for wearable health sensors (Figure 7f).

Song et al. prepared an in-plane deformation field sensor array based on ZnO thin film by the RF sputtering method [96]. With the doping of the lithium metal element in ZnO, the sensitivity of this device was 100 times higher than that of ordinary strain sensors. This can be attributed to the lower conductivity of the doped ZnO that reduced the shielding effect of the polarized charge and also prevented the mutual crosstalk between the individual sensing units, allowing each sensing unit to share the same highly sensitive film.

##### Substrate

The performance of PENG-based sensors depends mainly on the piezoelectric material. However, the substrate can also significantly affect its performance. Currently, the main suitable substrates used to improve the performance of devices are PET and PDMS.

Gao et al. proposed a BaTiO_3_-based flexible piezoelectric nanogenerator [97]. They adopted PET as the substrate of the device, which exhibited a higher output performance than the other substrates of the piezoelectric nanogenerator (Figure 7g). Owing to the excellent flexibility of PET, the device was deformed by the solution under the external force and thus efficiently converted mechanical energy into electrical energy (Figure 7h). Moreover, the insulating properties of PET reduced the energy loss in the process of the charge flow, thus improving the output performance of the device (Figure 7i).

Yan et al. prepared a BaTiO_3_-based flexible piezoelectric nanogenerator [98]. They selected polydimethylsiloxane (PDMS) as the substrate, and the output current of the device was up to 261.40 nA. The output of the device was increased by the excellent insulating property of PDMS, which reduced the energy loss during transmission. Furthermore, its flexibility protected the piezoelectric material from contamination and provided protection against mechanical damage to the device. Therefore, PET and PDMS are excellent candidates for the preparation of PENG-based sensor substrates. 

#### 3.3.2. Comfortability and Durability Enhancement Method

Owing to the long-term contact of the PENG-based non-invasive sensor with the wearer’s skin, its comfortability and durability are also very important. Generally, relatively rigid piezoelectric materials cannot establish a highly conformal contact with the skin and result in the discomfort of the wearer. Polymer piezoelectric materials and composite piezoelectric materials possess an excellent elasticity and flexibility, making them a favorable choice. Furthermore, the introduction of microstructures to the film surface in order to increase the contact area is also a promising option.

Siddiqui et al. fabricated a lead-free flexible PENG by the electrospinning technique [99]. The nanofibers in the device consisted of a composite of P(VDF-TrFE) and BaTiO_3_ nanoparticles (BT NPs), which were embedded in elastomeric PDMS to fabricate the PENG. When positioned inside the shoe, the elastomeric PDMS dissipated the applied force uniformly and avoided the pressure body’s direct contact with the soft polymeric fibers, thus reducing local damage and fragility and improving wearer comfort and the device’s durability.

**Figure 8:**
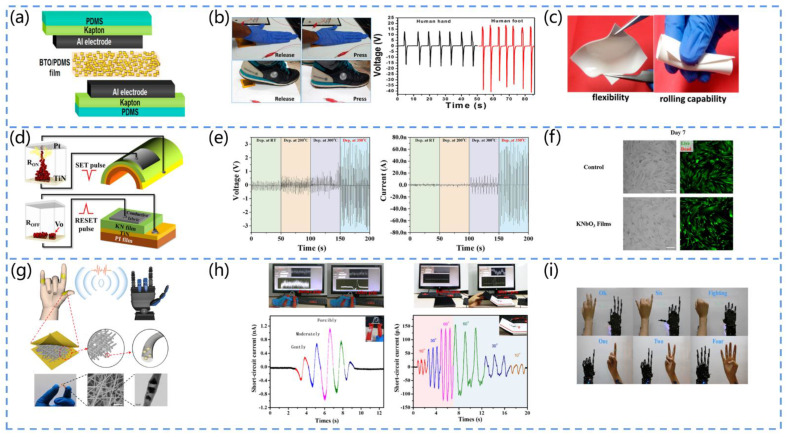
(**a**) Schematic diagram of the structure of the CPENG device. (**b**) Comparison of the voltages generated by the hand and foot release and the pressure applied to the CPENG. (**c**) Photo showing the excellent flexibility and rolling ability of the film. Reprinted with permission from [100]. Copyright 2017, ACS Publications. (**d**) Schematic diagram of the structure and operation of the KN-PENG. (**e**) Piezoelectric output generated by KN membranes formed at different temperatures used for the preparation of the PENG. (**f**) Comparison of the survival rates of human dermal fibroblasts on cell culture plates and on KNbO_3_ membranes over 7 days (Green and red colors indicate live and dead cells, respectively). Reprinted with permission from [101]. Copyright 2017, ACS Publications. (**g**) Structure of a self-powered piezoelectric sensor based on cowpea-structured PVDF/ZnO nanofibers. (**h**) Detection of the grip force and angle of book opening and closing by the piezoelectric current signal. (**i**) Application of sensors in gesture remote control. Reprinted with permission from [102]. Copyright 2019, Elsevier.

Alluri et al. developed a flexible, high-performance piezoelectric nanogenerator (Figure 8a) [100]. They prepared composite films (CFs) of BaTiO_3_ nanotubes (BTO NC) and polydimethylsiloxane (PDMS) by the solution casting technique. The device produced a higher power output (area power density of 7 mW/cm^2^) at a lower external force (988.2 Pa). It could be used in daily life to collect energy from human hand and foot movements, generating V_pp_ values of 29 V (palm) and 55.9 V (foot), respectively (Figure 8b). Furthermore, the device possessed the advantages of a light weight, flexibility, and durability, thus improving the wearer’s comfort (Figure 8c).

#### 3.3.3. Biocompatibility Assurance

Biocompatibility is a basic requirement for PENG non-invasive sensors. Although lead (Pb)-based ceramics such as PZT exhibit high electrical coefficients, their brittleness, rigidity, and toxicity limit their application in non-invasive sensing devices. Lead-free ceramics, such as KNbO_3_, NaNbO_3_, etc., have an excellent biocompatibility and can thus serve as PZT ceramic replacements. Furthermore, while most polymeric piezoelectric materials have an excellent biocompatibility, their piezoelectricity is relatively weak. The development of piezoelectric composites, which have the advantages of both organic and inorganic materials, could partially solve this problem.

Lee et al. fabricated a KNbO_3_ (KN)-based piezoelectric nanogenerator (Figure 8d) [101]. They grew amorphous-phase KNbO_3_ (KN) films on TiN/polyimide/poly (ethylene terephthalate) substrates at a low temperature (350 °C). The PENG exhibited an excellent output performance (~2.5 V, ~70 nA) under strain and strain rates of 0.76% and 0.79%/s, respectively (Figure 8e). Moreover, the KN films possessed an excellent reliability and biocompatibility, making the material an outstanding candidate for self-powered medical devices (Figure 8f).

Deng et al. proposed a self-powered piezoelectric sensor (PES) for the monitoring of pressure sensing and bending motion (Figure 8g) [102]. They fabricated cowpea-structured PVDF/ZnO nanofibers using an electrostatic spinning technique. The device had both the piezoelectric effect and polymer flexibility. Furthermore, it could work in both the compression and bending modes, with excellent flexibility, a high sensitivity, skin adaptation capacity, and mechanical stability (Figure 8i). Based on this, the authors implemented a self-powered gesture remote control system that can transmit the pulse signals from human fingers to the robot’s palm, showing promise for application in the field of medical monitoring (Figure 8h).

### 3.4. Applications of PENGs as Non-Invasive Sensors

PENGs have promoted the development of non-invasive flexible and wearable biosensors in recent years. Non-invasive flexible and wearable biosensors can accurately monitor variations in physiological characteristics to continuously track different health parameters in a patient-centric manner and provide valuable insights for healthcare applications.

#### 3.4.1. PENG-Based Biofluid Sensors

Biosensors are widely used for the non-invasive chemical analysis of biological fluids, such as saliva, sweat, urine, tears, and interstitial fluid (ISF). Biofluids can be sampled non-invasively, which means that this approach will not damage the outermost protective layer of the skin and lead to contact with the blood, resulting in a minimal risk of injury to the patient.

##### Saliva

Saliva is a rich complex biological substances composed of a large number of intracellular and extracellular components that permeate through the blood [103,104,105]. Therefore, saliva is an excellent non-invasive sample for blood analysis aiming to monitor the health status of the patient.

**Figure 9:**
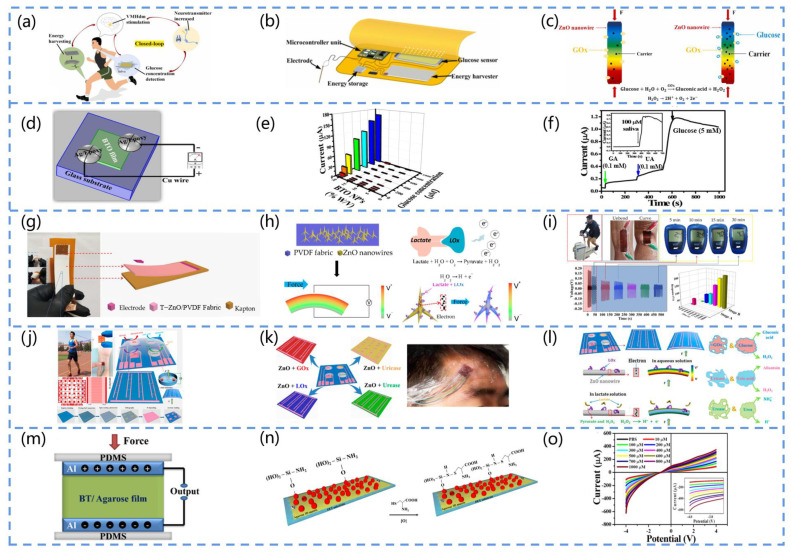
(**a**) Schematic diagram of the blood glucose monitoring sensor and brain–computer interface used to build a closed loop. (**b**) Components of the blood-glucose-sensing system. (**c**) Sensing mechanism of the device (*F* represents force). Reprinted with permission from [106]. Copyright 2022, Elsevier. (**d**) Schematic diagram of the BTO-NP-based sensor. (**e**) Three-dimensional graph of the current response of the BTO-NP-based glucose sensor at different concentrations (Glucose concentration are 50 μM~1 mM, BTO NP concentration are 5, 10, 15, 20% (*w*/*v*)). (**f**) *I-T* curves of glucose under interferences such as 0.1 mM galactose and 0.1 mM uric acid (Black arrows represent 5 mM glucose, green arrows represent 0.1 mM galactose, and blue arrows represent 0.1 mM uric acid). Reprinted with permission from [107]. Copyright 2016, Elsevier. (**g**) Schematic diagram of the structure of the self-powered biosensor based on T-ZnO/polyvinylidene fluoride (PVDF)/fabric. (**h**) Diagram of the working mechanism of the device (*V* represents voltmeter, the color of the rainbow indicates the magnitude of the output piezoelectric voltage). (**i**) Blood lactate concentration in the sweat of the test participant at different time points (5, 10, 15, 20, 25, and 30 min), stage A is the response of the biosensor in the phase from no sweating to sweating, stage B is the response of the biosensor to detect the sweat lactate concentration, F is force. Reprinted with permission from [109]. Copyright 2020, MDPI. (**j**) Structure and manufacturing process of self-powered non-invasive sensors. (**k**) Device’s adhesion to an exerciser for sweat analysis (Monitoring of lactate, glucose, uric acid and urea). (**l**) Sensing mechanism of self-powered non-invasive wearable devices (Red is Gox with glucose, blue is uricase with uric acid, and green is urease with urea). Reprinted with permission from [110]. Copyright 2017, ACS Publications. (**m**) Schematic structure of a BaTiO_3_/agarose (BT/Ag)-based PENG. (**n**) NH_2_-functionalized cysteine sensor. (**o**) *I-V* curve of the sensor detecting the cystine concentration (10–1000 µM). Reprinted with permission from [112]. Copyright 2017, Elsevier.

For example, Yang and his team fabricated a sensing system for the real-time monitoring of the blood glucose concentration in human saliva [106]. It can be worn on various parts of the human body, as illustrated in Figure 9a. Figure 9b presents the components of the system, which mainly include an energy harvester, a blood glucose sensor, a micro-control unit, an energy storage device, and electrodes. The sensing mechanism is attributed to the coupling of the enzymatic reaction and the piezoelectric effect, where GOx on the ZnO nanowires reacts enzymatically with glucose upon contact with the glucose solution, producing gluconic acid and hydrogen peroxide. Furthermore, as H_2_O_2_ decomposes, H^+^ can be adsorbed on the nanowire surface, and e^−^ is transferred to the Zno nanowire, increasing the surface carrier density (Figure 9c). This approach extends the application scope of integrated sensing systems in disease medicine. 

Selvarajan et al. developed a self-powered sensor based on BTO films for monitoring glucose in the saliva (Figure 9d) [107]. Figure 9e depicts the relationship between the sensor current response, BTO NP concentration, and glucose concentration. As expected, the 20% w/V BTo thin film sensor had a higher current response to the glucose concentrations ranging from 0 to 1 mM. Furthermore, 5 mM of glucose and 0.1 mM of interferent (galactose and uric acid) were used for the comparison, as shown by the I-T response curves in Figure 9f, indicating that the current response of the galactose and uric acid was negligible compared to that of glucose. This suggests that the sensing device is sensitive and has a wide linear concentration range and low detection limits, providing a suitable method for other relevant clinical applications.

##### Sweat

Sweat is an ideal sample for non-invasive biosensors, as it is readily available and offers a wealth of physiological information about the health status [108]. For example, lactic acid in sweat is an important metabolite of anaerobic glycolysis and can be used to determine tissue viability and fitness levels. Meanwhile, uric acid in sweat is a major metabolite of purines and can be used to detect diseases related to gout and leukemia. The metabolism of glucose in sweat is related to blood sugar and can be used to detect diabetes, while Na^+^ and K^+^ are related to osmolality. As a result, sweat can be applied to monitor an individual’s physical health and diagnose diseases.

Lactate is a major component of sweat, and its concentration can be used to assess an athlete’s maximum performance during high-intensity activity and provides an advance warning. In 2020, Mao et al. designed a portable PENG-based biosensor that can be used for sweat analysis [109], and the structure of the sensor is presented in Figure 9g. They adopted Kapton as a substrate for the device and modified the T-ZnO-nanostructured fabric with lactate oxidase (LOx) to achieve the real-time monitoring of the maximum lactate steady state (MLSS) of athletes in non-invasive conditions. According to Figure 9h, lactic acid reacts with the enzyme to produce pyruvate and H_2_O_2_, followed by the decomposition of H_2_O_2_ into H^+^, O_2_, and e^−^, and electrons are transferred to the T-ZnO nanowire array, increasing the surface carrier density. The magnitude of the output piezoelectric voltage is indicated by the colors of the rainbow, and the piezoelectric output is dependent on the mechanical energy provided by an external force. Figure 9i depicts a practical application of the sensor for MLSS monitoring. The results show that the monitoring of MLSS with this biosensor is feasible.

Additionally, the continuous detection of the above-mentioned sweat components is essential. Instead of conventional sweat sensors, PENG-based sweat sensors can be composed of several different enzyme-modified unit devices, enabling the continuous detection of multiple molecules. Han et al. designed a piezoelectric electronic skin that can be used for sweat analysis [110]. As presented in Figure 9j, the device can be attached to different parts of an athlete’s body to monitor their physiological status by analyzing the sweat composition. The device contains four piezoelectric biosensing units composed of ZnO nanowires modified by four enzymes, Lox (lactate oxidase), Gox (glucose oxidase), urease, and urease, which can monitor the glucose, lactate, urea, and uric acid concentrations in the sweat in real time (Figure 9k). Figure 9l presents the detailed working mechanism of the piezoelectric biosensing unit in this electronic skin. Its mechanism can be attributed to the piezo-enzyme response coupling effect of the enzyme/ZnO nanowires, with the piezoelectric pulses of the PENG providing the power and sensing data.

##### Urine

The method of monitoring urine through non-invasive techniques has numerous advantages, such as the protection of patient privacy and simplification of the process of collecting and handling samples. Cysteine in urine is an essential amino acid in the body that contains a thiol group. Its abnormalities are associated with chronic diseases such as Alzheimer’s-like diseases, Parkinson’s disease, and rheumatoid arthritis. Therefore, urine detection can be used for the routine analysis of diseases [111].

Selvarajan et al. fabricated a PENG-based cysteine sensor [112]. The device consists of an Agarose/BaTiO_3_-NH_2_ (Ag/BT-NH_2_)-film-based cysteine sensor in parallel with a BT/Ag-film-based piezoelectric nanogenerator (BT/Ag PENG), where the output power of the PENG is used to drive the sensor, as shown in Figure 9m. Figure 9n depicts the cysteine detection mechanism. In the presence of BT-NH, R-SH (thiol) loses an H^+^ and transforms into a sulfate, and the sulfate side chain (R-S) in cysteine is a strong nucleophilic reagent that reacts with the NH groups on the BT NPs (electrophilic reagents). This results in the nucleophilic attack of R-S^−^ on the NH group, resulting in the formation of an S-N bond. As a result, the cysteine binds to the film, increasing the surface’s negative charge. In addition, the sensor’s cysteine response is measured through the I-V technique, and the results indicate that the −4 V current increases with the increasing cysteine concentration (10 M–1 mM), and the increase in the current is linearly related to the cysteine concentration (Figure 9o). Compared to conventional sensors, this method enables the real-time analysis of urine samples using non-invasive methods and exhibits a comparable or higher performance, including an excellent sensitivity, selectivity, reproducibility, and stability.

#### 3.4.2. PENG-Based Respiratory Sensors

Breath plays an important role in human vital signs, and it can reflect changes in health conditions such as asthma, pneumonia, bronchitis, and other respiratory diseases [113,114]. In order to achieve the real-time monitoring and analysis of respiratory parameters, such as the molecules of some specific gases in exhaled air, respiratory mechanical signals, and temperature, various portable and miniaturized self-powered respiratory sensors have been developed.

##### Exhaled Gas Molecules

Previous research suggests that exhaled gas contains numerous organic and inorganic metabolites, some of which are closely related to human health and can serve as signals for certain diseases [115,116]. However, gas analysis instruments face numerous limitations, such as complex structures, high material costs, and unsustainable operation. Therefore, it is urgent to develop sensing devices that are portable, simple, and sustainable.

**Figure 10:**
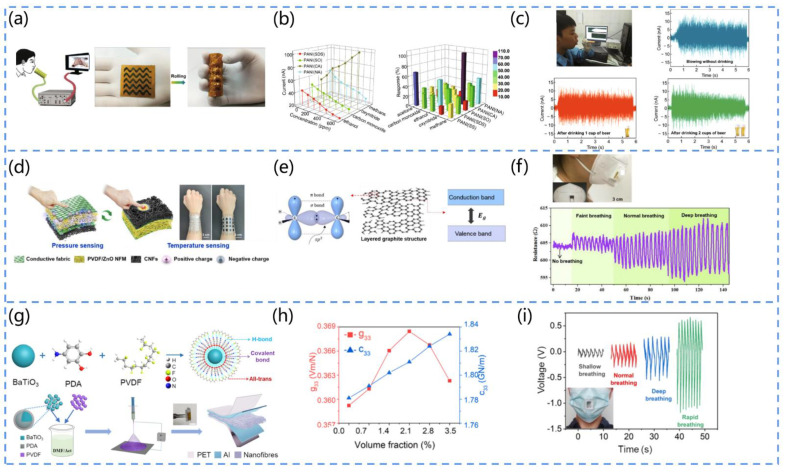
(**a**) Working principle and structure of PANI/PVDF-based piezoelectric sensors. (**b**) Relationship between the gas marker concentrations of each sensing unit. (**c**) Schematic diagram of fatty liver diagnosis by the monitoring of alcohol. Reprinted with permission from [117]. Copyright 2018, Springer Link. (**d**) Schematic diagram of the structure and sensing mechanism of the device. (**e**) Diagram of the preparation process of CNFs. (**f**) Output voltage at different frequencies of breathing. Reprinted with permission from [126]. Copyright 2021, Elsevier. (**g**) Schematic diagram of the process of piezoelectric fabrics modified with polydopamine. (**h**) Piezoelectric voltage coefficient and stiffness coefficient of piezoelectric fabrics with different PDA contents. (**i**) Schematic diagram of the output of piezoelectric fabrics with different breathing modes. Reprinted with permission from [132] Copyright 2021, Elsevier.

Fu et al. designed a non-invasive, self-powered breath analyzer [117]. Figure 10a shows the working mechanism and structural schematic of the device, in which the core component is an aniline/polyvinylidene fluoride (PANI/PVDF) film. The piezoelectric effect induced by the PVDF on the gas flow can provide power, and the gas-sensitive properties of the PANI electrode can be used for the detection of exhaled gas. The authors used sodium sulfate, sodium dodecyl benzene sulphonates, sodium oxalate, camphor sulphonic acid, and nitric acid as dopant sources of five polyaniline derivatives to form five gas-sensing units, which can diagnose respiratory diseases such as asthma, diabetes, cirrhosis, and inflammation via the detection of exhaled gases (acetone, ethanol, CO, NO_x_, and CH_a_) in the concentration range of 0 to 600 ppm, and the results show an excellent sensing performance (Figure 10b). In addition, the device can analyze ethanol gas, thus simulating the diagnosis of fatty liver (Figure 10c).

Oximetry is a key indicator that is used to assess the presence of respiratory failure in patients with COVID-19 [118,119,120,121,122]. Lin et al. proposed a self-powered exhalation oxygen-sensing mouthpiece for the real-time monitoring of lung health information [123]. They used tetrapod ZnO (T-ZnO) hybridized with polyvinylidene fluoride (PVDF) attached to a flexible fabric. Owing to the coupling of the gas-sensitive properties with the piezoelectric effect, the T-ZnO/PVDF-based sensor converts the exhaled airflow energy into a piezoelectric signal, which not only serves as a power source but also increases with the increase in the exhaled oxygen concentration, thus monitoring the exhaled oxygen concentration in real time and reflecting the oxygen-filling capacity of the human lungs. This self-powered wearable sensing device can facilitate the non-invasive health monitoring of lung diseases.

Exhaled ammonia is one of the biomarkers of impaired renal function. Some studies indicate that an ammonia concentration of 1.1 ppm is considered relatively healthy, while ammonia above 1.6 ppm is unhealthy [124]. The testing of ammonia concentrations in patients’ breath can be used to screen patients for potential kidney disease and provide effective treatment in advance. Zhang et al. created a PENG-based ammonia gas sensor [125]. The device consists of a flexible MoS_2_ sheet PENG and an Au-MoSe_2_-sensing membrane. The PENG attached to the human body can collect energy from various body movements to drive the ammonia sensor.

In conclusion, conventional gas-sensing devices have many limitations. The PENG-based self-powered respiratory sensor has attracted great interest from scholars in the field of medical sensing, and its electrical signal is closely related to the respiratory parameters, an aspect which has important clinical significance for the early diagnosis and treatment of numerous diseases.

##### Respiratory Temperature and Humidity

Except for exhaled gas molecules, human breathing involves heat and water. By analyzing the changes in the exhalation temperature and humidity, it is easy to distinguish four types of breathing: no breathing, weak breathing, normal breathing, and deep breathing. Furthermore, when a patient has symptoms of fever or respiratory inflammation, the exhaled gas temperature is always higher than that of a healthy patient. These characteristics enable electronic devices that can monitor breathing to have great potential for intelligent medical diagnostic applications.

Wang et al. developed an electronic skin textile that can precisely sense the breathing temperature [126]. The textile can be comfortably applied to human skin, as seen in Figure 10d. It consists of a piezoelectric polyvinylidene fluoride nanofiber membrane doped with zinc oxide nanoparticles (PVDF/ZnO NFM) and flexible heat-resistant carbon nanofibers (CNFs). The temperature-sensing principle is depicted in Figure 10e. The CNFs consist of disordered graphite structural layers, where each carbon atom is hybridized in the form of sp^2^, and the lone electrons that are not involved in the hybridization overlap with each other to form off-domain π-bonds. These off-domain electrons move freely in the carbon atomic plane. As the temperature increases, the electrons originally bound by the atoms gain energy and become free electrons, resulting in a decrease in the resistance of the CNFs. To demonstrate the ability of the sensing textile to monitor temperature changes in physical objects, the textile is attached to a mask, and the results indicate that the resistance of the textile varies with the temperature, thus enabling the monitoring of the temperature of exhaled air under different breathing conditions (Figure 10f). In addition, its temperature resolution is 0.381% /°C, and the temperature range is from 25 °C to 100 °C. This electronic skin textile has great promise for application in the diagnosis and monitoring of respiratory diseases. 

The absorption of water molecules can significantly enhance the electrical conductivity of nanofibers (NFs) [127]. Gu et al. developed an active humidity sensor based on lead-free NaNbO_3_ [128]. They synthesized a NaNbO_3_ piezoelectric nanofiber with a monoclinic chalcogenide structure by the far-field electrostatic spinning method and transferred it to a polymer substrate in order to fabricate a flexible active humidity sensor. The device has an output voltage of up to 2 V and a negative correlation with ambient humidity in the range of 5%~80%. Its humidity-sensing mechanism originates from the proton hopping between H_3_O^+^ groups driven by the piezoelectric potential, which increases the leakage current inside the NFs.

Vivekananthan et al. designed a piezoelectric nanogenerator (PENG)-driven biopolymer humidity sensor [129]. They coated piezoelectric collagen on cotton fabric with an upright structure and piezoelectric properties, enabling its use as an energy harvester and humidity sensor. The PENG-based sensor exhibits an excellent linear response in the 50–90% humidity range. Its sensing mechanism is the chemisorption of water vapor on the collagen membrane surface or the replacement of oxygen on the membrane surface. This work provides a new idea for the development of non-invasive medical systems, novel electronic skin, and self-powered sensors.

##### Respiratory Mechanical Signal

Research has shown that the respiratory rate and intensity provide a wealth of physiological information about the health status, which can be applied for exercise monitoring and disease warning [130]. Several scholars have investigated the association of the respiratory rate with physical health status. For example, it may originate from anesthetic or sedation overdose and elevated intracranial pressure during surgery when the respiratory rate is lower than 0.2 Hz (12 bpm). Meanwhile, when the respiratory rate is greater than 0.4 Hz (24 bpm), it may derive from diseases such as anemia, fever, heart failure, and hyperthyroidism. Currently, it is necessary for people to develop self-powered electronic devices for monitoring the respiratory rate in order to monitor non-invasive diseases of the respiratory system, especially during the periods of outbreaks of COVID-19, aggravated air pollution, and frequent hazy weather [131].

Su et al. designed a polydopamine-modified nonwoven piezoelectric fabric (PMNP) [132]. They extensively researched barium-titanate-doped polyvinylidene fluoride (BTO/PVDF) piezoelectric nanofibers by inserting polydopamine (PDA) between BTO nanoparticles and PVDF to form an interfacial layer, as shown in Figure 10g. According to Figure 10h, PDA can considerably contribute to the local all-trans conformation and modulus matching at the nanofiller–polymer interface. This is shown by the strong dependence of the piezoelectric voltage coefficient (g_33_) and stiffness coefficient (c_33_) on the volume fraction of PDA, which reaches a maximum at 2.15%. In the study, PMNP textiles were sewn onto the mask to evaluate the device’s sensing capabilities. It was found that the exhaled airflow deforms the PMNP fastened to the mask, resulting in output signal waveforms with various intervals and amplitudes for various breathing patterns, allowing for the precise and quick differentiation of various breathing patterns (Figure 10i). For example, shallow breathing leads to a slow deformation of the PMNP, resulting in a smaller amplitude and longer interval of the electrical signal.

Liu et al. proposed an active sensor based on PVDF piezoelectric nanogenerators for respiratory sensing and healthcare monitoring [133]. The output voltage and output current during respiration were as high as 1.5 V and 400 nA, respectively, which corresponded to the physiological signals, demonstrating the excellent accuracy and reliability of the device. Furthermore, the authors simulated several rapid respirations and compared them to the normal respiratory rate, and these respiratory profiles provided detailed information on the kinetics of the respiratory process and have great significance for the development of sensing devices for lung function assessment.

Wang et al. created a wearable, multifunctional piezoelectric MEMS (micro-electro-mechanical system) device [134]. The device measures less than 10 mm when placed on the finger and can be positioned in a mask to monitor movement states such as resting, running, and walking based on the breathing rate. The principle of the device is based on the voltage generated by the inverse piezoelectric effect of the PZT material to obtain the respiratory rate. When the mask is worn for a long time, an early lung health warning can be obtained based on an abnormally fast or slow breathing rate.

From the above, we discover that researchers have developed a wealth of PENG-based non-invasive respiratory monitoring devices. They are positioned on the body, including the chest, abdomen, and throat, to collect mechanical signals. The direct contact of these areas with the skin ensures that PENG-based respiratory sensors can be driven by the physical motion caused by breathing and collect respiratory signals through this process. However, their operation has certain limitations and is vulnerable to environmental influences, such as humidity and electromagnetism. Other equipment, such as nasal airflow sensors, is also not reliable. In conclusion, PENG-based sensors offer a new way to monitor breathing patterns and sleep quality.

#### 3.4.3. PENG-Based Cardiovascular Sensors

Cardiovascular diseases, including heart and blood vessel diseases, have become one of the biggest threats to the health of the elderly [135,136]. Fortunately, with the advent of various advanced medical sensors, most diseases can be diagnosed in advance. The real-time and continuous monitoring of the body’s physiological signals, such as the radial pulse, heart rate, respiratory rate, and blood pressure, can provide medical information about many cardiovascular states and thus prevent some cardiovascular diseases.

##### Pulse

The radial artery pulse can be detected on the wrist. Its amplitude, frequency, and waveform are important cardiovascular parameters. Self-powered continuous arterial pulse monitoring systems can improve the quality of treatments for cardiovascular disease, thus extending the lives of patients. Traditional pulse monitoring sensors exhibit significant power consumption drawbacks that limit their continuous operation. To overcome this limitation, non-invasive sensors based on piezoelectric nanogenerators are used for the continuous monitoring of arterial pulses.

**Figure 11:**
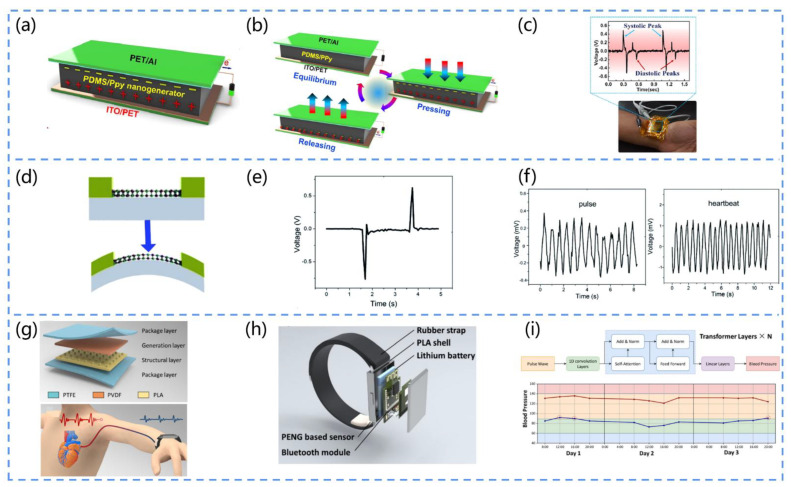
(**a**) Schematic diagram of the structure of the PDMS/PPy-based PENG. (**b**) Diagram of the operating mechanism of the PDMS/PPy-based PENG. (**c**) The voltage response of the PDMS/PPy-based PENG as arterial pulse pressure, the blue arrow represents the peak systolic pulse and the red arrow represents the peak diastolic pulse. Reprinted with permission from [137]. Copyright 2022, Elsevier. (**d**) Schematic diagram of the structure of the SnSe-based PENG whilst bent (The arrows represent the direction force action). (**e**) The output voltage of the SnSe-based PENG reaches 760 mV. (**f**) The heart rate is detected with the device attached to the wrist and chest. Reprinted with permission from [139]. Copyright 2021, the Royal Society of Chemistry. (**g**) Material and sensing schematic of BPPW. (**h**) Component modules of BPPW. (**i**) Deep learning model construction of BPPW and application for the prediction of blood pressure among test subjects (The blue line represents the patient’s blood pressure and the dotted line represents the moment when it exceeds the normal value). Reprinted with permission from [141]. Copyright 2022, MDPI.

In 2022, Veeralingam et al. designed a Pb-free, sensitive, and flexible piezoelectric nanogenerator for measuring the human arterial pulse pressure [137]. They adopted a rotational coating technique to prepare polydimethylsiloxane/polypyrrole piezoelectrically active materials. Then, they deposited the PDMs/PPy composite polymer onto an ITO-coated PET substrate and coated an aluminum film onto the PET substrate as a counter electrode (Figure 11a). The mechanism of the PENG is shown in Figure 11b. When external pressure is present, nano-dipoles are formed inside the PDMS/PPy piezoelectric composite film due to the synergistic effect of the PDMS and αβ’-PPy polymers, generating piezoelectric potential. When the external pressure is released, the generated nano-dipoles are neutralized, and the piezoelectric potential disappears. This periodic compression and release of pressure to the PENG produce an AC-type response. Once the device is mounted on the wrist, the pressure response of the arterial pulse to the PENG can be observed. The high-intensity peak can be attributed to the systolic pressure peak of the pulse, while the low-intensity peak can be attributed to the diastolic pressure peak of the pulse (Figure 11c). This PENG opens up new avenues for the development of biosensors for medical diagnostics.

Karan et al. developed spider-silk (SS)-based bio-piezoelectric nanogenerators (SSBPENG) [138]. Naturally, abundant spider silk exhibits a vertical piezoelectric coefficient of up to ~0.36 pm/V, excellent mechanical properties, an excellent biocompatibility, and biodegradability. The prepared SSBPENG devices show a high output performance, energy conversion efficiency (up to 66%), and sensitivity to arterial pulses (signals generated by tension). Furthermore, they can enable the health monitoring of throat movements during coughing, speaking, and drinking. Thus, these bio-piezoelectric materials can be used to fabricate PENGs for the future development of the medical field.

##### Heart Rate

The application of non-invasive sensors in the medical and healthcare fields can significantly improve the quality of life and living standards of patients and reduce healthcare costs. Medical sensors that measure the heart rate may be used for the detection of abnormal conditions, such as bradycardia or tachycardia.

Li and his team proposed a SnSe piezoelectric nanogenerator that can non-invasively monitor important health signs, such as the heart rate (Figure 11d) [139]. They adopted a mechanical stripping method to obtain a SnSe that exhibits a strong anisotropic piezoelectric response and piezoelectric output voltages reaching up to 760 mV (Figure 11e). By integrating the SnSe nanogenerator and a single MoS-based sensor, they obtained a self-powered sensor unit (SPSU) without the need for a power supply. The device was installed on the wrist and chest as a pulse sensor, respectively, and the measurements of the heart rate (~90/min) were consistent, exhibiting an excellent sensing performance (Figure 11f). Furthermore, the SnSe piezoelectric nanogenerator could successfully convert the mechanical energy generated by the heartbeat into electrical energy. Therefore, SnSe piezoelectric nanogenerators have great potential in the field of healthcare.

In addition, Li et al. developed a MoSe_2_-based piezoelectric nanogenerator that can non-invasively monitor the human heart rate [140]. They synthesized a monolayer MoSe_2_ sheet by chemical vapor deposition and achieved a 50% larger piezoelectric output signal than MoS_2_ at a 0.6% strain, which indicates the excellent piezoelectricity of MoSe_2_. By connecting the MoSe_2_ nanogenerator to the tester’s chest, the heart rate could be monitored by V-t curves. The piezoelectric signals associated with the heartbeat, such as the frequency and amplitude, were significantly increased by high-intensity exercise, indicating its high sensing performance.

##### Blood Pressure

Blood pressure measurement is an important method for diagnosing hypertension, and it is classified into two categories: invasive and non-invasive. Invasive measurements are required for critical conditions and complex cases. Traditional non-invasive measurement methods include cuff compressions, such as mercury sphygmomanometers used in hospitals and electronic sphygmomanometers used at home. The human blood pressure constantly fluctuates; therefore, the development of sensors that continuously measure blood pressure can provide doctors with a relatively accurate foundation for diagnosis and enable them to avoid misdiagnosis.

Tan et al. fabricated a PENG-based smart wristband that can be applied for the non-invasive monitoring of blood pressure (Figure 11g) [141]. The device consists of PENG-based sensor, rubber band, PLA housing, Bluetooth module, and battery (Figure 11h). Moreover, the device can be used to acquire pulse wave signal signals with a noise ratio of 29.7 dB. As illustrated in Figure 11i, the principle of the device is to combine pulse wave data collected by a deep learning model with a pre-built regression model to predict the blood pressure. Wearing this wristband enables the continuous monitoring of a patient’s blood pressure, which opens up new ideas for the prevention and treatment of hypertension.

#### 3.4.4. PENG-Based Motion Sensors

##### Foot and Hand Movement

The movement monitoring of both the upper and lower extremities is in high demand in a variety of application scenarios, ranging from patient rehabilitation to exercise training. Exercises such as finger flexion, arm swinging, clapping, running, and jumping can improve blood circulation to various organs of the body, thereby improving overall health.

**Figure 12:**
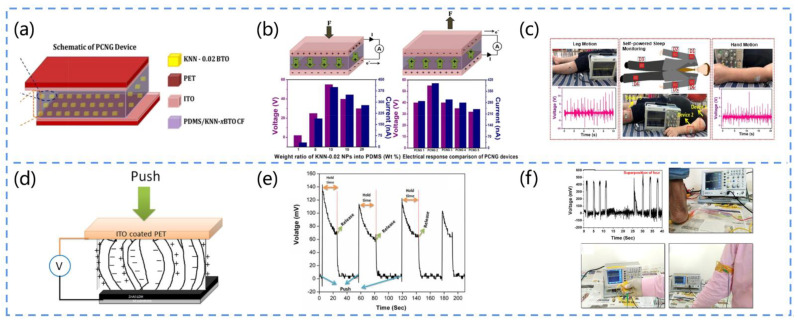
(**a**) Schematic diagram of the structure of the PCNG. (**b**) Schematic diagram of the device’s operating mechanism, output, and piezoelectric response (*I* represents current, *A* represents ammeter, *F* represents force). (**c**) The device monitors leg and hand movements during sleep. Reprinted with permission from [142]. Copyright 2019, Elsevier. (**d**) Schematic diagram of the structure of the PENG (*V* represents voltmeter). (**e**) Voltage pulses generated by the device under the action of an external force. (**f**) Photo of the piezoelectric output of four nanogenerators connected in series and the application of the device for muscle stretch testing. Reprinted with permission from [146]. Copyright 2020, Elsevier.

Vivekananthan developed a Pb-free flexible PENG (Figure 12a) [142]. They adopted a solid-phase reaction method to prepare Pb-free (1−x) KNaNbO_3_-x BaTiO_3_ nanoparticles (x = 0.02, 0.04, 0.06, and 0.08). Then, these particles were impregnated into a polydimethylsiloxane (PDMS) matrix to fabricate composite films for the piezoelectric nanogenerators. The doping of BTO enhanced the piezoelectric properties of the KNN material (maximum electrical output of 58 V, 450 nA) without affecting the orthogonal phase of KNN (Figure 12b). Furthermore, by attaching the PENG to the human hand and leg, it could be used as an active sensor to monitor human sleep and movement (Figure 12c). This work can help us to diagnose sleep disorders among the elderly and may also provide a new strategy for their mobile health monitoring.

Choudhry et al. designed a shoe insole nanogenerator (SING) [143]. They dispersed four common piezoelectric materials, BaTiO_3_ (BT), ZnO (ZO), soft pzt (P1), and hard pzt (P2), in silicon (Si) and then selectively added graphene nanopowder (GNP) to fabricate the SING and analyze its piezoelectric performance. In addition, to evaluate the sensing performance of the device, they conducted tests including foot stomping, walking, running and touching, in which the SING exhibited a high output performance (power density, 402 mW/m) in the case of real-time walking. The results suggest that this SING can be used as a self-powered biosensor for wearable devices, autonomous physiological monitoring, and tactile sensing.

Dutta et al. synthesized NiO@SiO_2_/PVDF nanocomposites and exhibited the application of this material for the energy harvesting of human motion and tactile electronic skin sensing [144]. They coated silica onto NiO nanoparticles, thus hindering the agglomeration of the NiO nanoparticles in the PVDF matrix, and the uniform dispersion of the nanofillers enhanced the piezoelectric and dielectric properties of the composites. As a result, the PENG composed of this material exhibited a high output (power density, 685 W/m^3^) and excellent conversion efficiency (13.86%), and it could light up to 85 LEDs when the PENG was gently touched by hand and charge a 2.2 μF capacitor to 22 V within 450 s. The device can not only power human-related electronic devices but also precisely detect an individual at rest or in motion. For example, the self-powered electronic skin sensor can be attached to a glove to distinguish between the movements of different fingers. This simple, sensitive, and portable piezoelectric nanogenerator opens up a new platform for the application of human-motion-based energy harvesters.

Kumar et al. proposed a piezoelectric nanogenerator based on a P(VDF-TrFE)/ZnO nanofiber membrane [145]. They dispersed ZnO in P(VDF-TrFE) solution at a concentration of 18% (*w*/*w*) and synthesized the P(VDF-TrFE)/ZnO nanocomposite by the electrostatic spinning method. The addition of the ZnO nanofiller significantly improved the phase fraction, roughness, viscosity, dynamic modulus, and loss modulus of the solution and reduced the damping coefficient of the solution. Moreover, they characterized the composite film, and the results showed that the addition of ZnO nanoparticles enhanced the β phase and improved the activity and sensitivity of the composite film. Moreover, the PENG could record the hand motions such as hand pressure, bending, and tapping with the voltage output. This PENG provides a simple self-powered pathway for many medical health-monitoring devices.

##### Muscle Stretching

Manjula et al. developed a flexible ZnO-nanosheet-based piezoelectric nanogenerator (Figure 12d) [146]. They prepared ZnO nanosheets by adopting a low-temperature, single-step hydrothermal method. Conductive aluminum foil was the substrate used for growing the ZnO nanosheets. Since the aluminum foil and ZnO nanosheets formed a layered double hydroxide (LDH) layer, the substrate provided excellent adhesion for the growth of the ZnO nanosheets, without any additional seed layer deposition. This PENG was tested under real-time mechanical forces such as muscle stretching, finger tapping, and foot pressure to verify the output of the nanogenerator and confirm the piezoelectric voltage (Figure 12e). Connecting this device in series with four 400 mV devices further enhanced the output voltage and confirmed the high stability and repeatability (400 mV) of the piezoelectric nanogenerator (Figure 12f). This work is useful for the future bioenergy harvesting and monitoring of human mechanical motion signals.

##### Eyelid Movement

Long-term work of the eyes and brain can cause eye strain. Eye fatigue may cause serious health problems if it is not detected and treated in a timely manner. Lü et al. developed an ultra-thin piezoelectric sensor that can monitor eye fatigue by collecting information about blinking, such as a high blink frequency, long eye closure time, weak gaze strength, and other abnormal conditions [147]. They adopted lead zirconium titanate (PZT) nanoribbons as the functional material for the sensor. Owing to the high piezoelectric coefficient of PZT, it can generate a high voltage signal when mechanically deformed by external forces. Moreover, the thickness of the sensor is only 10 µm, which allows for better contact with the eyelid skin during blinking. This study contributes to the assessment of eyestrain and the prevention of health problems.

#### 3.4.5. Other Devices

In cases of poor wound healing, wound infection is a major concern. For example, it is not uncommon for a small wound to result in systemic infection or even amputation, especially in immunocompromised individuals. Even in healthy individuals, the appearance of a wound after trauma or surgery can be a major challenge. As a result, various wound dressing methods have been developed, such as electrical stimulation, which has been widely accepted. Due to its simplicity, portability, and biocompatibility, the PENG is an excellent candidate that can be used to assist in wound healing.

**Figure 13:**
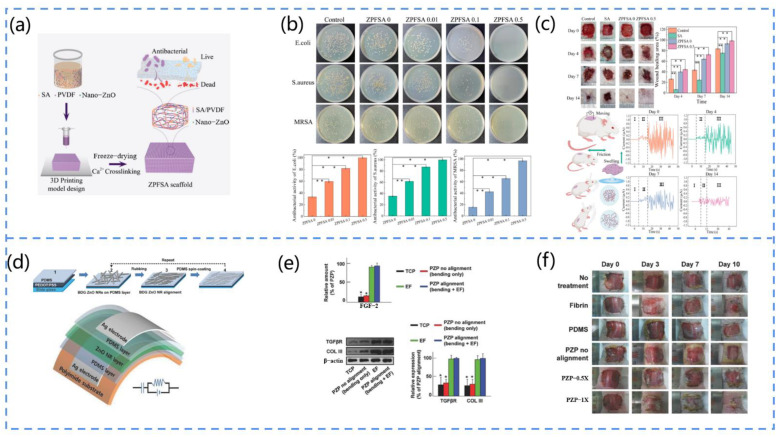
(**a**) Schematic diagram of the fabrication and composition of the ZPFSA piezoelectric scaffold. (**b**) Diagram of the test of the resistance of the device to different bacteria, subplots are images of scaffolds with E. coli, S. aureus, and MRSA and their antimicrobial properties, respectively, ** denotes *p* < 0.01. (**c**) Diagram of the relationship between the time and area of trauma healing and body surface current (*A* is an ammeter; Ⅰ is baseline; Ⅱ is current on the normal skin; Ⅲ is current on the wound). Reprinted with permission from [148]. Copyright 2022, ACS Publications. (**d**) Illustration of the manufacturing process and structure of PZP (1, 2, 3, 4 represent the four steps of BDG ZnO NR-based PZP preparation). (**e**) Diagram of the gene expression of PZP-promoted cell growth factor, * *p* < 0.05 versus with EF or PZP alignment. (**f**) Diagram of the process of PZP in promoting wound healing (PZP-0.5X denotes nine-layer PZP with a filling density of 54.8%, PZP-1X denotes nine-layer PZP with a filling density of 95.2%) [149]. Copyright 2017, Wiley Online Library.

Liang et al. developed a ZnO-modified PVDF/sodium alginate (SA) piezoelectric hydrogel scaffold (ZPFSA) that can serve as a trauma dressing for skin repair (Figure 13a) [148]. The scaffold is prepared by 3D printing technology with a dual piezoelectric release model of vertical expansion and horizontal friction, exhibiting a stable piezoelectric response, favorable biocompatibility, and excellent antibacterial properties. In this device, SA acts as a support to drive the swelling and stretching of the PVDF in the absorption of the traumatic exudate (Figure 13b). The ZnO-modified PVDF has stable hydrophilic polarization and shows good antimicrobial properties, and the ordered and regular pore structure of PVDF can rapidly absorb and remove exudate, thus generating a stable model and suitable piezoelectric current to stimulate cell proliferation, orderly collagen deposition, and trauma healing (Figure 13c). Ultimately, in the study, it accelerated the healing rate of rat wounds and prevented scar formation. This work suggests that piezoelectric trauma dressings have promising applications in the field of skin repair.

Bhang et al. designed a ZnO-based piezoelectric skin patch (PZP) (Figure 13d) [149]. The fabricated patch consists mainly of PDMS and ZnO nanorods, which can generate pulsed potentials and EF after deformation under external force to promote skin recovery. The authors attached the PZP to the trauma sites of rats, and 54.8% and 95.2% of the ZnO-based patches produced 320 and 900 mV voltage outputs, respectively (Figure 13e). The range of 0.150 to 1.2 V was found to be the ideal voltage for accelerating wound healing. In the authors’ in vitro experiments, the curved patches created EF, which also encouraged dermal fibroblast migration and increased the generation and gene expression of fibroblast growth factors. Animal studies have revealed the significant effectiveness of zinc-oxide-based piezoelectric patches (Figure 13f).

Du et al. created a biological patch (HPSP) composed of shellfish hydrogel (PDA-PAAm) and electro-spun polyvinylidene-fluoride (PVDF) nanofiber-based PENG that can be used to accelerate skin wound healing [150]. The patch mimics the action of the shellfish adhesion proteins and trauma endogenous electric field (EF), which can attach to the wound surface and generate a low-frequency pulsed voltage, thus facilitating the in vitro promotion of fibroblast proliferation and migration and even partial hair follicle regeneration. The test results showed that HPSP reduced the wound closure time of the skin defects by about 1/3, which is a significant advance in wound healing.

Although wound-monitoring sensing devices are promising, there are still some difficulties limiting their actual clinical use. First, the size of the PENG should be customized to match the size of the wound. Then, biocompatibility, elasticity, and durability are necessary for the PENG’s ability to produce electrical stimulation. In addition, the effects of body fluids should be prevented. Wound exudate and body fluids can corrode PENG devices, and the question of how to maintain a high energy conversion efficiency and long-term stability in this environment is an issue that requires careful consideration. Finally, wound healing is a complex and dynamic process, and wounds of different sizes and depths require electrical stimulation of different intensities to promote wound healing.

The characteristics and performance of numerous PENG-based non-invasive sensors are listed in Table 1. The wearing position of a non-invasive sensor has a great impact on energy harvesting, owing to the fact that the wearing position determines whether it is driven by motion or by the respiratory airflow. The sensors in positions such as the hands, feet, throat, abdomen, and chest are primarily motion-driven. As these positions are more active and vigorous, they commonly exhibit a higher output performance than other sensors. The monitoring of temperature, humidity, exhaled gas, and biofluids is achieved by monitoring systems placed around the mouth, nose, forehead, and skin. These sensing systems can directly access biomarkers and obtain more biological information; thus, their sensing performance is comparatively stronger. Moreover, smaller devices possess greater portability and can be placed in more locations. Considering their long-term wearability, the materials chosen for consideration are generally those with a better biocompatibility. Furthermore, the performance of PENG-based sensors is outstanding in terms of the detection range and sensitivity. Compared to conventional sensors, these sensors can be self-powered and exhibit a better selectivity and sensitivity due to the recognition of specific molecules by the active layer.

**Table 1:** Summary of recent PENG-based non-invasive sensing systems for the collection of medical information.

Wear Position	Target	Main Material	Size	Output Performance	Sensing Performance ^a^		Ref.
					Detection Range	Selectivity	
Around the mouth	Saliva	GOx/ZnO	3 × 1 cm^2^	0.16 V	0~4.1 mg/L	Good	[106]
		BTO/PVA/PVDF	2.5 × 2.5 cm^2^	900 nA, 5 V (11 N, 11 Hz)	0.1 μM~1 mM	Good	[107]
On the forehead and hand	Sweat	T-ZnO/PVDF/LOx	14 × 14 cm^2^	0.446 V	0~8 mmol/L	—	[109]
		ZnO/Kapton/Enzyme	—	72.4, 57.8, 72.8, and 57.6 mV	0~20 mM/L	Good	[110]
On the skin	Urine	Ag/BT-NH_2_	3 × 3 cm^2^	80 V, 285 nA	10 µM–1 mM	Good	[112]
Around the nose and mouth	Oxygen	T-ZnO/PVDF	4 × 2 cm^2^	0.195 V	10%~20%	—	[123]
	Multi-exhaled gas	PANI/PVDF	5 × 5 cm^2^ × 10 μm	104.6 nA	0~600 ppm	Good	[117]
	Ammonia	Au-MoSe_2_/MoS_2_/PDMS	—	26 mV	10~100 ppm	Good	[125]
Around the nose and throat	Temperature	PAN/ZnO/PVDF	3 × 1.5 cm^2^	1.3 V	25 °C~100 °C	—	[126]
Around the nose, mouth, and throat	Humidity	Collagen/Al/PP	3 × 3 cm^2^	45 V, 250 nA	50–90%	Good	[129]
		NaNbO_3_/PDMS	2.0 × 1.0 × 0.2 cm^3^	2 V	5%~80%	Good	[128]
Around the nose, mouth, and throat	Respiratory rate	PVDF/Au	42 × 20 × 0.6 mm^3^	1.5 V, 400 nA	0~50 bpm	—	[133]
		PZT/SiO_2_	—	12 mV	0~45 times/min	—	[134]
		PDA/BTO/PVDF	3 × 3 cm^2^	9 V (0.74 N)	—	—	[132]
Around the wrist	Pulse	PET/PDMS/PPy	—	12 V, 0.11 µA	0~72 times/min	—	[137]
		SS/PDMS	25 × 20 mm^2^	21.3 V, 0.68 µA	—	—	[138]
On the chest	Heart rate	SnSe/PET/MoS_2_	—	760 mV, 28 mW m^−2^	0~90 times/min	—	[139]
		SnSe/PET/MoS_2_	—	60 mV	0~60 times/min	—	[140]
Around the wrist	Blood pressure	PLA/PTFE/PVDF	4.8 × 2.8 × 2 cm^3^	0.41 V, 0.21 µA	40~160 mmHg	—	[141]
On the fingers, arms, and legs	Leg and hand motion	KNN-x BTO/PDMS	3 × 3 cm^2^	58 V, 450 nA	—	—	[142]
		Ceramic NPs/Graphene/Silicone	5 × 7 × 0.7 cm^3^	27 V, 429.23 µA	—	—	[143]
		NiO/SiO_2_/PVDF	1.6 × 2.5 cm^2^	53 V, 0.3 µA	0.1~1.2 kPa	—	[144]
On the arms and legs	Muscle stretching	ZnO/ITO/PET	—	0.1 V	—	—	[146]
On the eyelid	Eyelid motion	PZT/PI/Kapton	—	0.2 V	0~1.8 mm	—	[147]
On the skin	Wound	PVDF/ PDA-PAAm hydrogel	1 × 1 cm^2^ × 100 μm	0.85 V, 40 nA	—	—	[150]
		PVDF/ZnO/SA	1.5 × 1.5 cm^2^ × 3 mm	1.29 µA	—	—	[148]

^a^ “—”in the table implies that the data were not recorded in the research.

## 4. Conclusions and Prospects

This review comprehensively summarized the research progress in PENG-based sensors and their potential applications in non-invasive medical devices. On the one hand, the wide selection of different piezoelectric materials and sensing strategies have brought great opportunities to PENG-based sensors for health monitoring, performance enhancement, and disease diagnosis and prevention. On the other hand, although the independent and continuous monitoring and detection of mechanical movements and metabolites of the human body have been realized, there remains an unmet medical need to further tailor the design of PENG-based medical sensor systems to practical applications. Some probable drawbacks relate to the power supply, and some relate to the non-invasive sensors. Future work may focus on the following directions. For instance, a PENG-based sensing system may be coupled with other functions or integrated with other modules to enable intelligibility and facilitate the rapid transmission of medical diagnostic data. Meanwhile, changing the raw material of the device could improve the accuracy of the sensing and reduce the cost of the device. Furthermore, the introduction of new packaging technologies and protective materials could mitigate the wear and aging of the device. Considering the complexity of the in vitro operating conditions, the challenges and urgent issues that remain to be addressed in tapping into the practical applications of PENG-based medical sensing systems are listed below.

### 4.1. Power Supply

#### 4.1.1. Energy Conversion Efficiency

The present most urgent problem associated with PENGs is their relatively low energy conversion efficiency. The strategy used to improve the efficiency of piezoelectric energy harvesting is mainly to optimize the circuit management [151]. Piezoelectric energy harvesters usually contain an AC-DC converter or a two-stage conversion circuit or use nonlinear techniques, such as the synchronized switch harvesting on inductor (SSHI) technique and synchronous electrical charge extraction (SECE).

The following circuits can be mentioned: AC-DC piezoelectric-energy-harvesting circuits, which rectify the AC power generated by piezoelectric transducers; two-stage piezoelectric-energy-harvesting circuits, which maximize the rectification power; SSHI technology, which eliminates the effect of capacitive terms and increases the output power; and SECE technology, which is load-independent compared to other technologies. However, these circuits also have their own drawbacks; therefore, it is possible to design a suitable circuit according to the requirements of the practical applications.

#### 4.1.2. Lifetime

A PENG-based sensor that monitors the human disease requires long-term wear by the patient; thus, its service life is important. Device aging, abrasion, weak connections, human metabolites, and airborne dust can affect the service life of PENG-based sensors and reduce their sensing and output performance, which can be solved by adopting new packaging technologies. The introduction of fatigue and aging tests can contribute to the evaluation of the device’s lifetime.

Furthermore, the development of self-healing PENGs is a promising direction. Materials with self-healing properties mainly contain piezoelectric composites [152] and piezoelectric molecular crystals [153]. PENGs fabricated from these materials can effectively avoid the fatigue and fracture of piezoelectric materials caused by periodic external forces, thus extending the device’s life.

### 4.2. Non-Invasive Sensors

#### 4.2.1. Bio-Inspired Properties

Bio-inspired properties such as biocompatibility and biodegradability broaden the scope of the current applications of PENG-based sensors, which is promising for the non-invasive diagnosis and treatment of patients.

Previously, we discussed biocompatibility, and the main solution is to select a composite material with excellent biocompatible properties to fabricate PENGs that ensure compatibility while possessing strong piezoelectric properties. For biodegradability, the candidate materials that can be applied include natural polymers, such as silk, cellulose, chitosan, plants, and fish skin. Furthermore, biodegradable metals such as Mg and Mo can also be used. Although some synthetic polymers are also biodegradable, natural polymers are an optimal choice in terms of the cost, degradability, and sustainability. In the future, long-term clinical studies of biocompatibility and degradability could be performed with animals and combined with recent insights into human adverse reactions. This would facilitate the further optimization of piezoelectric devices.

#### 4.2.2. Sensitivity

Owing to the diversity of human movement patterns and the rich variety of metabolic substances, it is a great challenge to accurately diagnose diseases from the large number of abundant physiological signals. The search and development of high-precision piezoelectric sensing materials is an important solution. 

Implementing microstructures placed on substrates is an excellent method for achieving a high sensitivity. Micro-dome, micro-pyramidal, and micro-pillar structures can be used to categorize the surface microstructures of sensing devices. In addition to these typical three-dimensional geometric microstructures, certain bionic-inspired substrate architectures have also been created. Furthermore, we can strive to create bespoke high-precision sensor devices in order to reduce the overall structural error by electrostatic spinning, in situ polymerization, chemical vapor deposition, and other approaches. Additionally, increasing the sensor’s stability can enable the sensing performance to be maintained for a long time.

#### 4.2.3. Anti-Interference

Since PENG-based medical sensing systems need to operate in the complex environment of the physical surface, it is important that the devices are immune to interference. For example, sensors that rely on the temperature and humidity of exhaled gases are more susceptible to external influences. 

Optimizing the encapsulation strategy of a PENG is a feasible approach, such as the coating of the surface of the device with a layer of flexible, stretchable polymer to prevent the wearer’s biofluids from penetrating the PENG, which is beneficial in improving its interference resistance [154,155]. Furthermore, optimized signal processing through the use of oscilloscopes, digital filters, low-noise current, and voltage amplifiers, to name a few examples, and signal management modules for wireless signal transmission systems may also improve the signal-to-noise ratio of the system.

#### 4.2.4. Multifunction

Numerous PENG-based sensors have been created for disease detection. However, disease diagnosis requires access to information involving many aspects of the human body, and a single functional medical sensor may be insufficient for disease diagnosis. Moreover, the condition and individual variability of each patient may lead to unreliable diagnostic results and challenge the accuracy of disease diagnosis. Achieving the multi-functionality of PENG-based sensing systems can enhance the performance of the devices and broaden their application scope. In principle, it is possible to introduce other functional materials, such as pyroelectric, electromagnetic shielding, and optoelectronic materials, into the piezoelectric material to provide PENG-based sensors with multiple functions. Meanwhile, integration with wireless transmission systems and artificial intelligence databases could improve the accuracy of disease diagnosis results and increase the convenience of wireless PENG-based sensors in the future.

## Data Availability

Data are available on request from the authors.
